# Supplementary material for: Mapping cis- and trans-regulatory target genes of human-specific deletions
Source: Nat Commun. 2025 Dec 20;16:11380. doi: 10.1038/s41467-025-67424-x (PMC12727812; doi:10.1038/s41467-025-67424-x)
Supplement: Supplementary file 1 — Supplementary Information [file 41467_2025_67424_MOESM1_ESM.pdf]

Supplementary Information

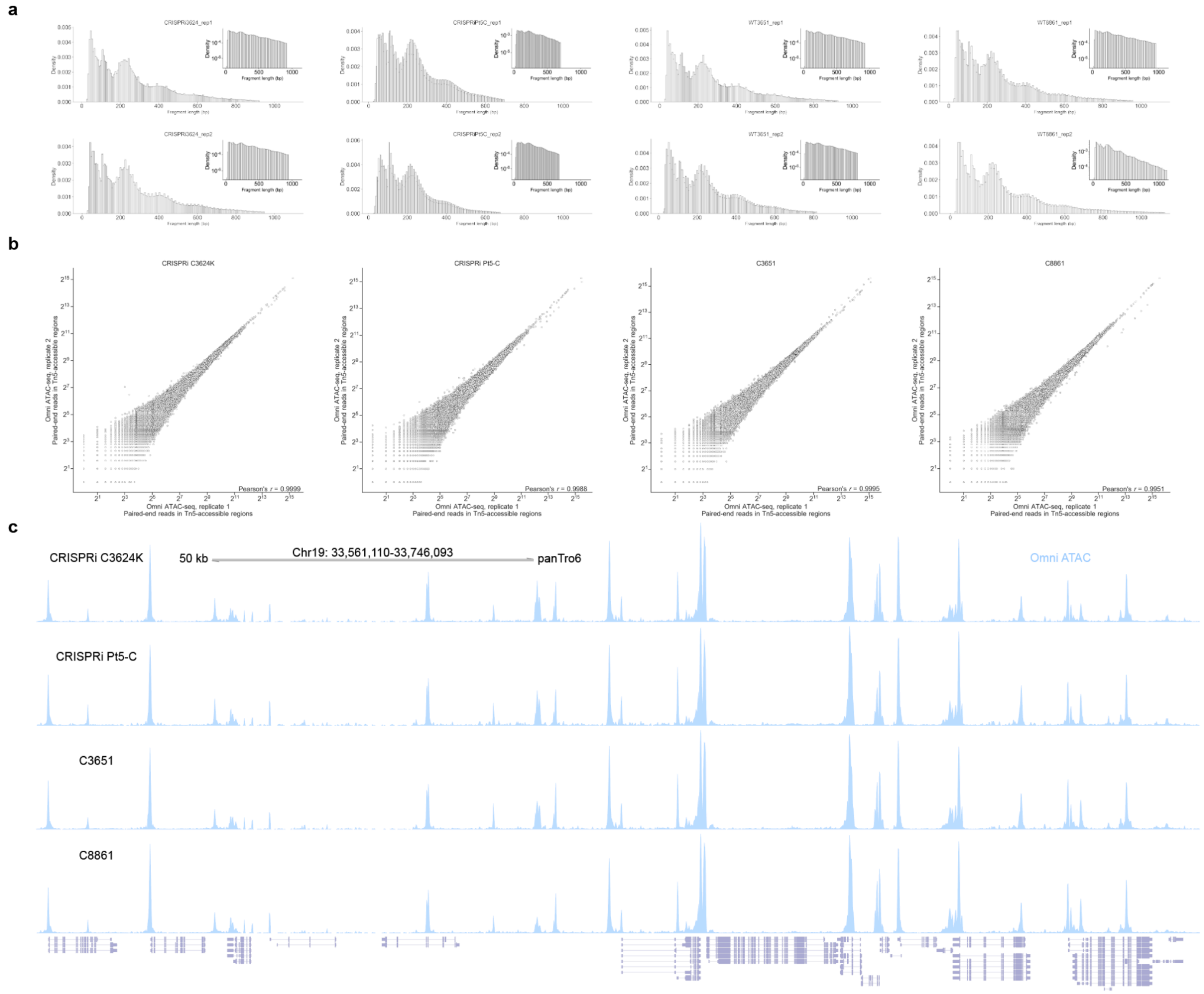

Supplementary Fig. 1 Omni ATAC-seq of chimpanzee iPS cells.

a, Distribution of Omni ATAC-seq fragment sizes. Inset: log-transformed histogram.

b, Correlation of reads within Tn5-accessible regions for Omni ATAC-seq technical replicates. Each point represents a Tn5-accessible region in any of the four iPS cells lines (5% FDR).

c, Omni ATAC-seq across a 184 kb region of the chimpanzee reference genome (panTro6).

a

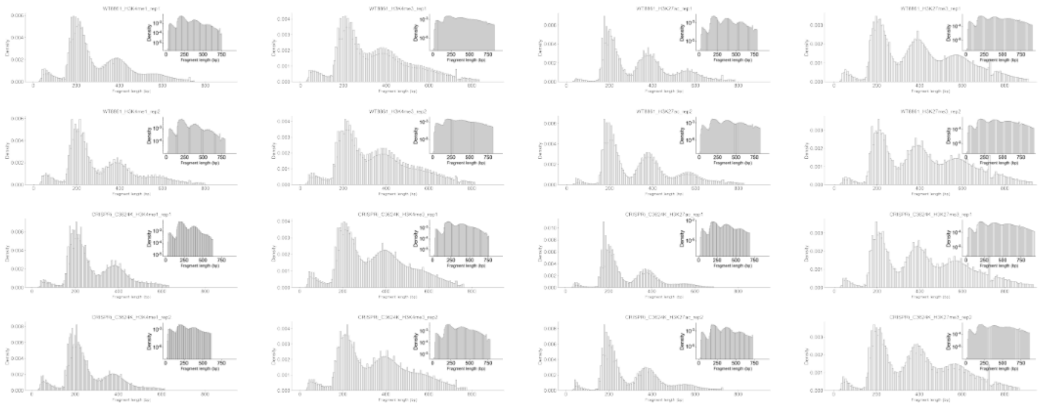

b

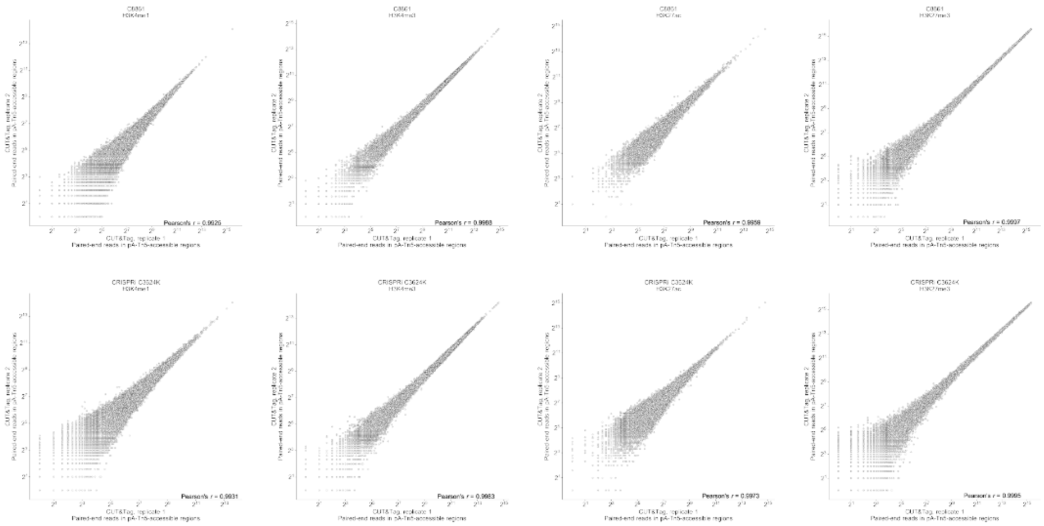

c

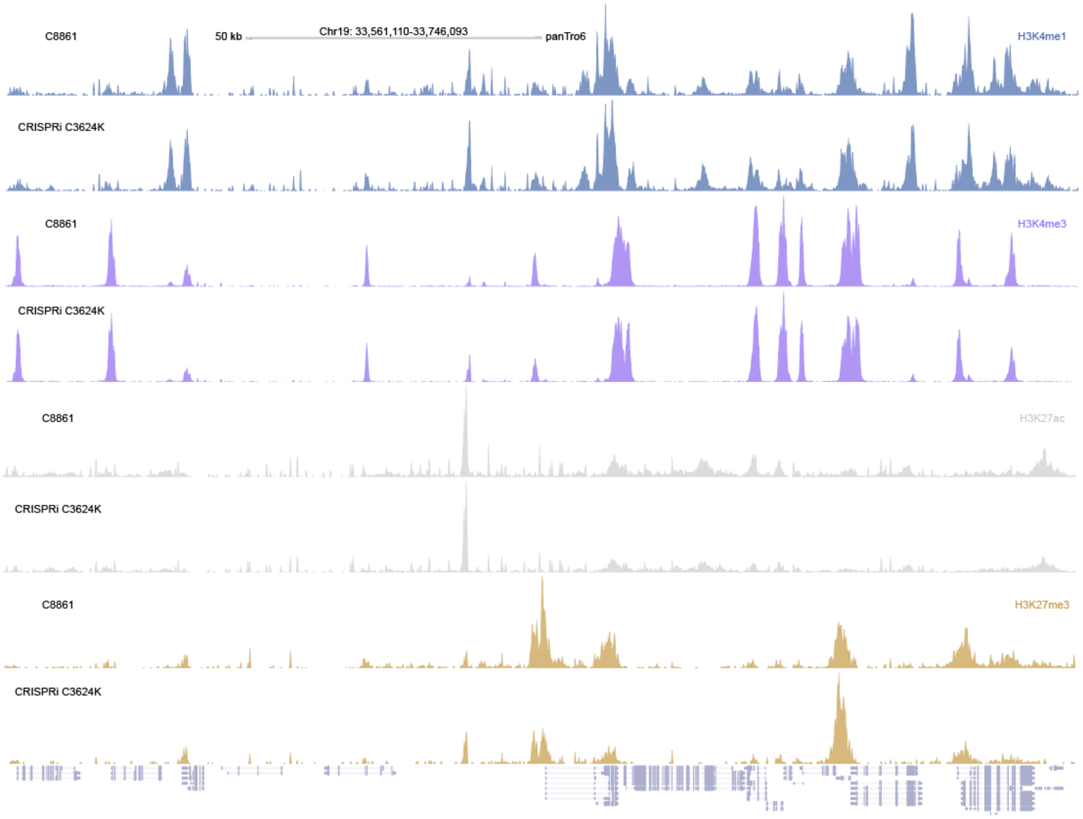
C8861
50 kb
Chr19: 33,561,110-33,746,093
panTro6
H3K4me1
CRISPRi C3624K
C8861
H3K4me3
CRISPRi C3624K
C8861
H3K27ac
CRISPRi C3624K
C8861
H3K27me3
CRISPRi C3624K

Supplementary Fig. 2 Profiling histone modifications with CUT&Tag of chimpanzee iPS cells.

a, Distribution of CUT&Tag fragment sizes for H3K4me1^{ab8895}, H3K4me3^{ab8580}, H3K27ac^{ab4729}, and H3K27me3^{9733S}. Inset: log-transformed histogram.

b, Correlation of reads within pA-Tn5-accessible regions for CUT&Tag technical replicates. Each point represents a pA-Tn5-accessible region in any of the two iPS cells lines (5% FDR).

c, CUT&Tag across a 184 kb region of the chimpanzee reference genome (panTro6).

a

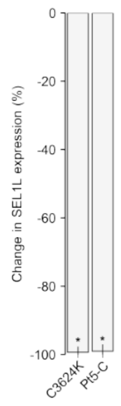

b

hDel-v1 sublibrary	Number of sgRNAs
hDels	156,820
Flanking hDels	14,084
Essential genes	8,068 (2,017 genes)
Representation-increasing genes	1,692 (423 genes)
Chimpanzee organoid-expressed genes	15,189 (5,063 genes)
Non-targeting controls	3,000
Total	198,853 (7,503 genes)

c

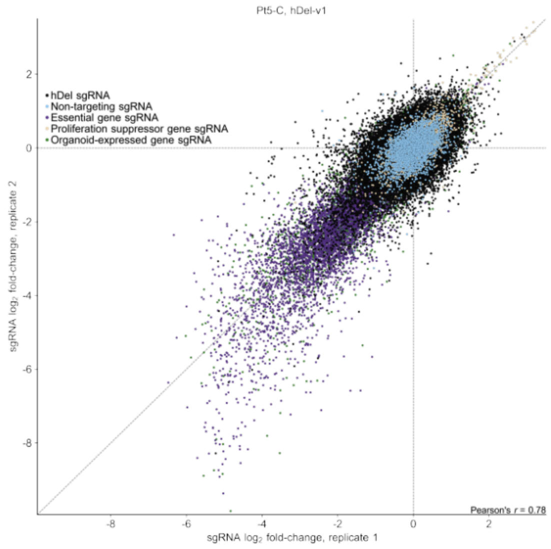

d

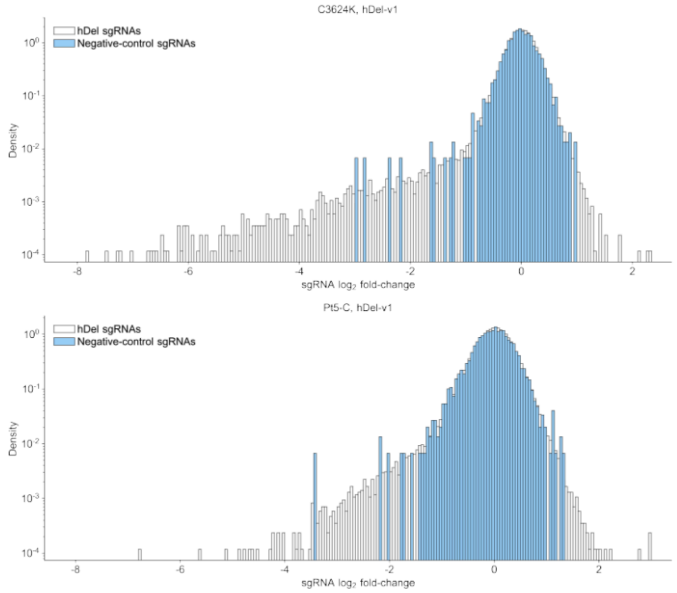

e

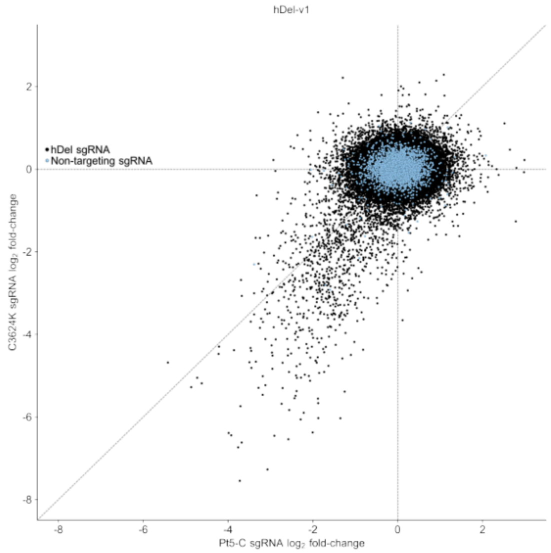

f

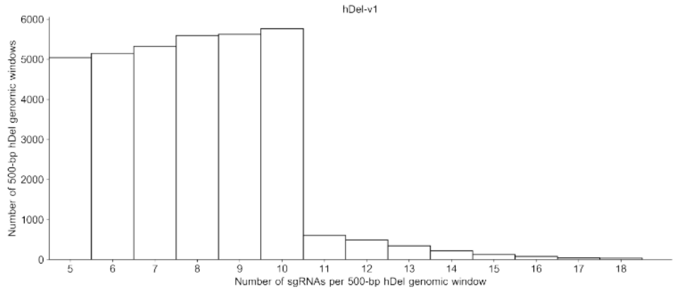

g

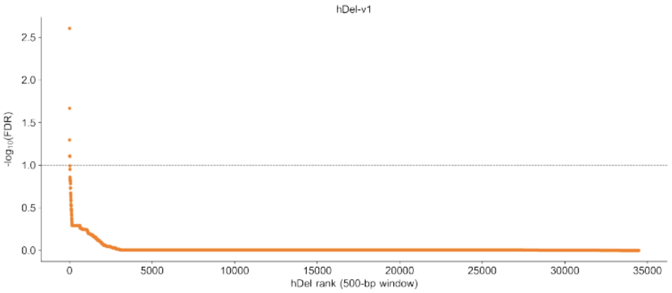

Supplementary Fig. 3 hDel-v1 CRISPRi-based genetic screens.

a, Change in *SEL1L* expression for chimpanzee CRISPRi iPS cells (C3624K, Pt5-C) harboring *SEL1L*-targeting sgRNAs compared to non-targeting sgRNAs as assessed by RT-qPCR.
b, hDel-v1 sublibraries.
c, Scatterplot of sgRNA $\log_2$ fold-change for hDel-v1 technical replicates in Pt5-C.
d, Distribution of sgRNA $\log_2$ fold-change for hDel-targeting and non-targeting sgRNAs in C3624K (top) and Pt5-C (bottom).
e, Scatterplot of sgRNA $\log_2$ fold-change for hDel-targeting and non-targeting sgRNAs in C3624K and Pt5-C.
f, Distribution of the number of sgRNAs per 500-bp hDel genomic window.
g, 500-bp hDel genomic windows ranked by α-RRA Benjamini-Hochberg-adjusted *p*-value.

a

hDel-v2 sublibrary	Number of sgRNAs
hDels	54,754
Flanking hDels	23,516
Essential genes	600 (98 genes)
Representation-increasing genes	394 (60 genes)
Non-targeting controls	2,000
Total	81,264 (158 genes)

b

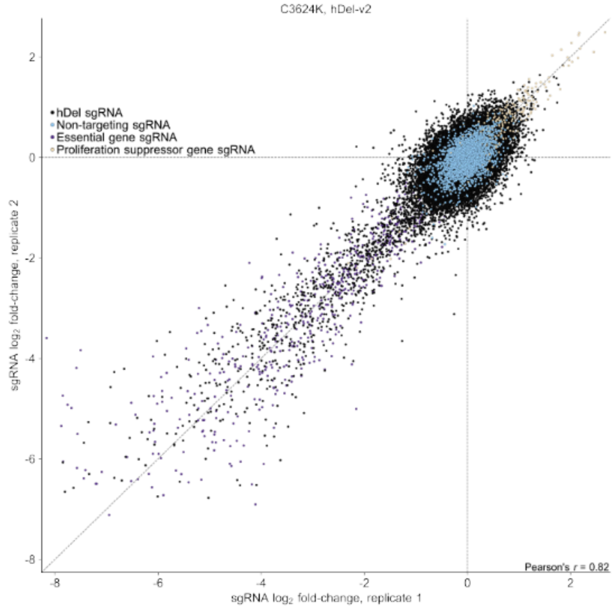

c

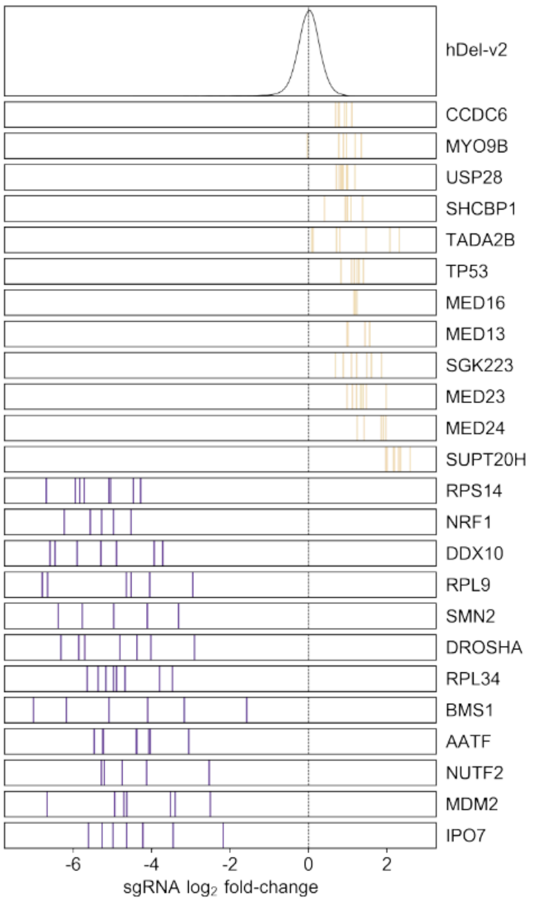

d

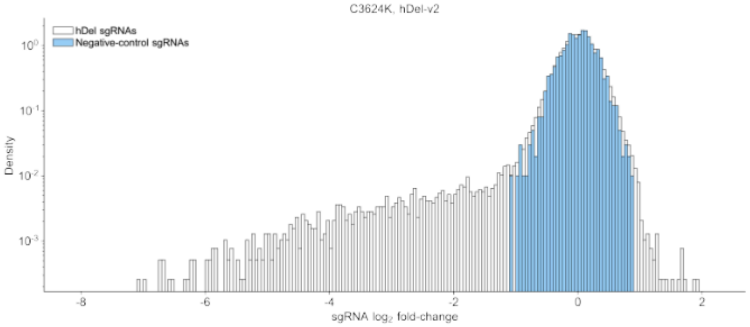

e

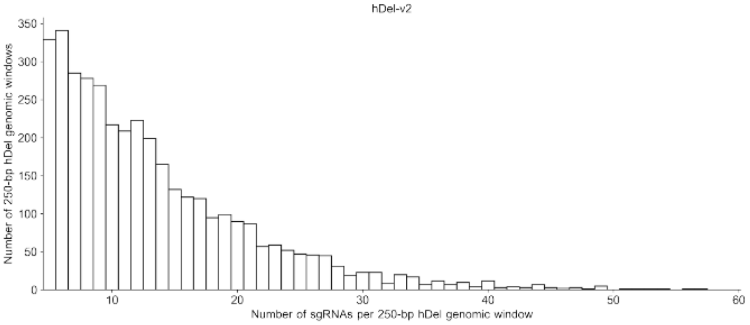

f

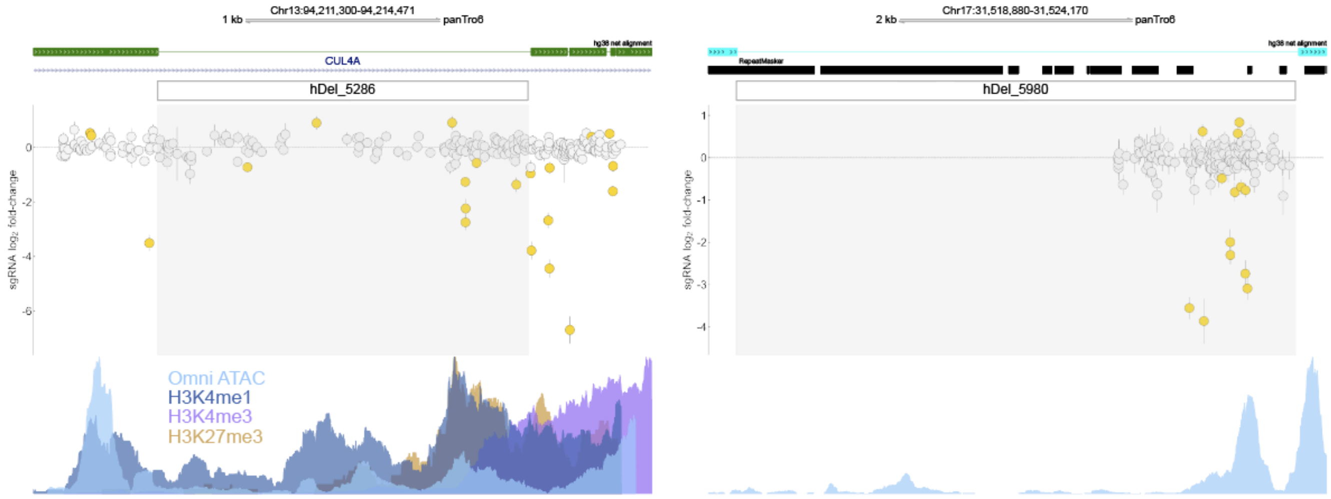

Supplementary Fig. 4 hDel-v2 CRISPRi-based genetic screen.

a, hDel-v2 sublibraries.

b, Scatterplot of sgRNA $\log_2$ fold-change for hDel-v2 technical replicates in C3624K.

c, Rug plot of sgRNA $\log_2$ fold-change for the 12 most enriched (khaki) and depleted (purple) genes as ranked by average sgRNA $\log_2$ fold-change. Each vertical line represents a transcription start site-targeting sgRNA.

d, Distribution of sgRNA $\log_2$ fold-change for hDel-targeting and non-targeting sgRNAs in C3624K.

e, Distribution of the number of sgRNAs per 250-bp hDel genomic window.

f, 250-bp hDel genomic windows intersecting epigenetic features. hDel_5286 (left) and hDel_5980 (right).

a

<u>hDel-v3 sublibrary</u>	<u>Number of sgRNAs</u>
hDels	122 (19 hDels)
Transcription start site	18 (12 genes)
K562 *cis*-regulatory element	10 (5 *cis*-regulatory elements)
Non-targeting controls	10
Total	160

b

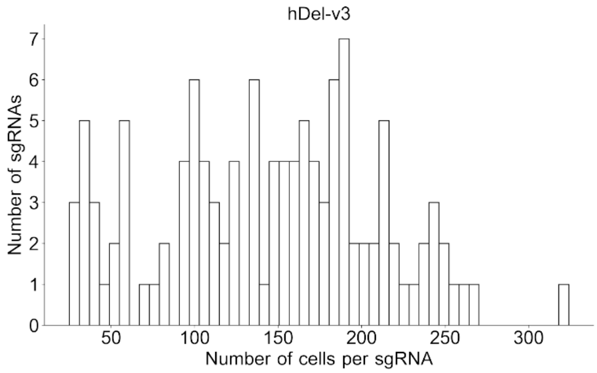

c

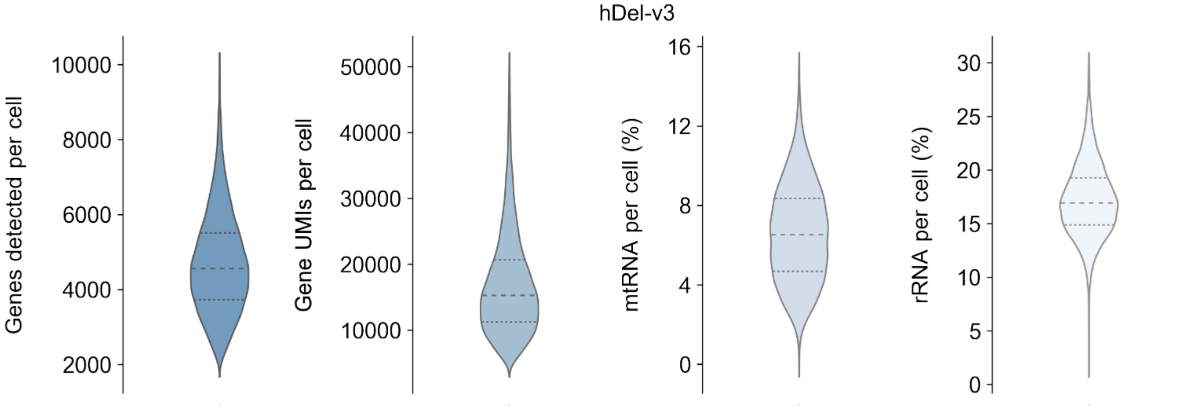

d

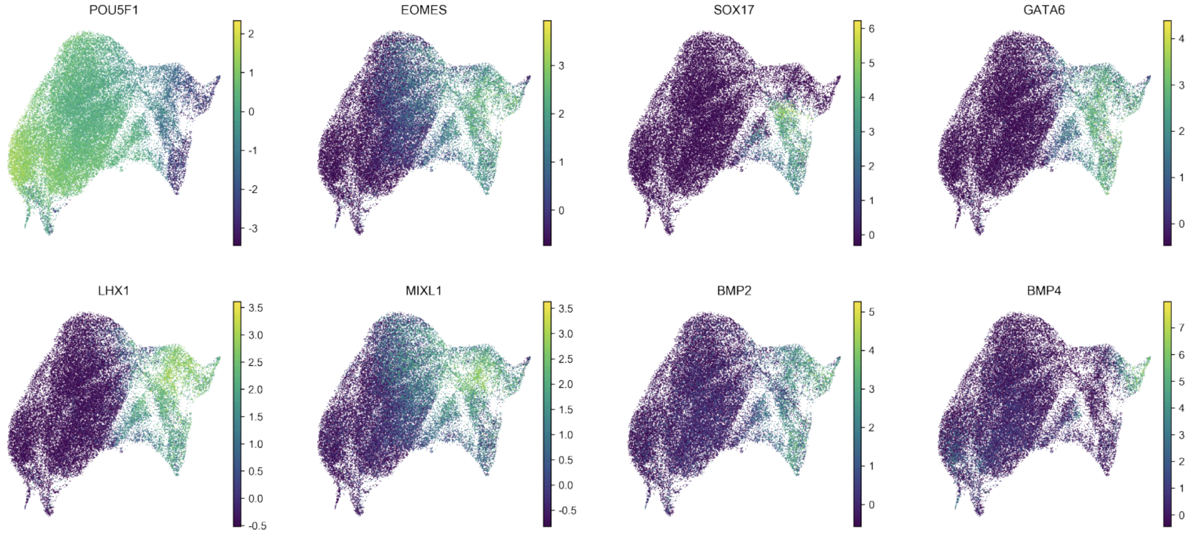

Supplementary Fig. 5 hDel-v3 Direct-capture Perturb-seq.

a, hDel-v3 sublibraries.

b, Distribution of the number of cells per sgRNA.

c, Distribution of the number of genes detected (far left), gene UMIs (center left), percent mtRNA (center right), and percent rRNA (far right) per cell.

d, UMAP projections colored by the normalized, log-transformed, and scaled expression of the indicated genes.

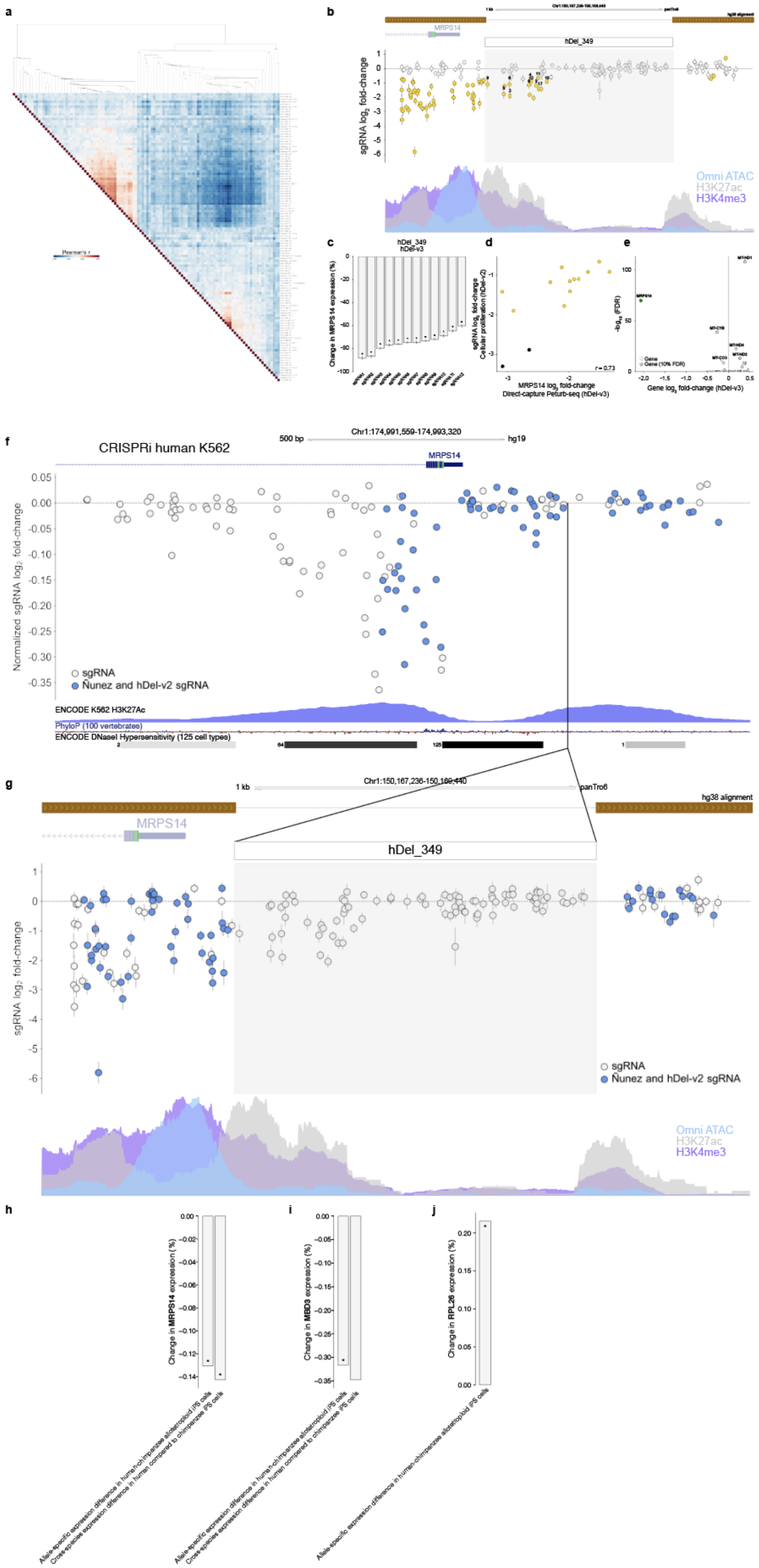

Supplementary Fig. 6 hDel-v3 *trans* differential expression and hDel_349.
a, hDel-v3 sublibraries.

b, Heatmap of Pearson correlations of gene $\log_2$ fold-change and hierarchical clustering for differentially expressed genes. The union of any gene differentially expressed (*n* = 2,864 genes, FDR < 0.1) in a sgRNA 'pseudobulk' (rows) is used for Pearson correlations and clustering.
c, hDel_349-targeting sgRNA $\log_2$ fold-change (hDel-v2; gold, FDR < 0.05) and *MRPS14* Omni-ATAC seq, H3K4me3, and H3K27ac in C3624K.
d, Differential *MRPS14* expression for cells harboring the indicated hDel_349-targeting sgRNA (*FDR < 0.1).
e, Scatterplot of hDel_349-targeting sgRNA $\log_2$ fold-change (cellular proliferation, hDel-v2) and *MRPS14* $\log_2$ fold-change (gene expression, hDel-v3).
f, *MRPS14* TSS-targeting sgRNA $\log_2$ fold-change in human K562 cells[22] and ENCODE K562 H3K27ac, PhyloP, and ENCODE DNaseI hypersensitivity. sgRNAs in blue are in the Nuñez et al. and hDel-v2 sgRNA libraries.
g, hDel_349-targeting sgRNA $\log_2$ fold-change (hDel-v2) and *MRPS14* Omni-ATAC seq, H3K4me3, and H3K27ac in chimpanzee iPS cells (C3624K). sgRNAs in blue are in the Nuñez et al. and hDel-v2 sgRNA libraries.
h-j, Comparing *MRPS14* (**h**), *MBD3* (**i**), and *RPL26* (**j**) expression from human and chimpanzee alleles. Gene expression change < 0 indicates reduced expression from human alleles.

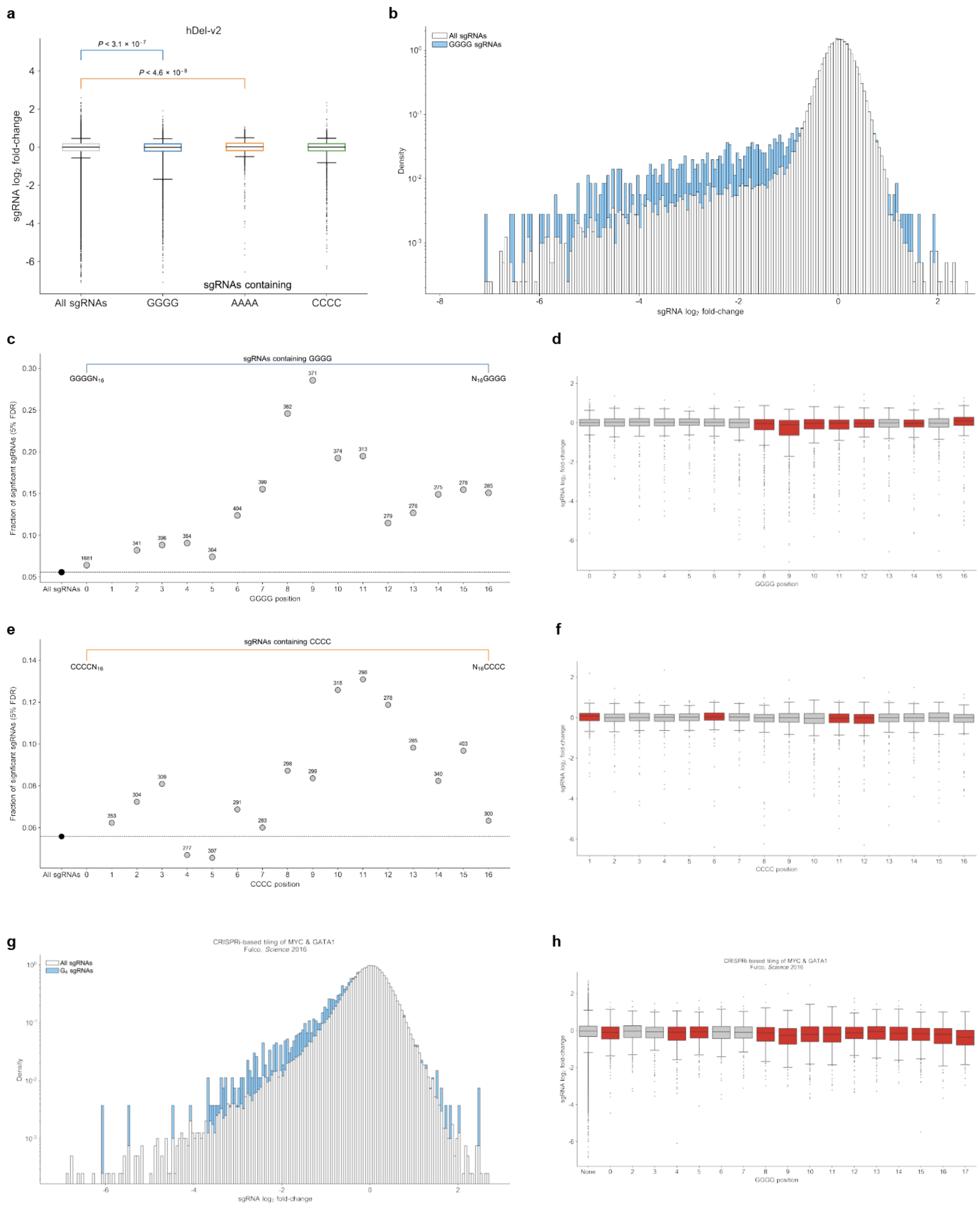

Supplementary Fig.7 sgRNA nucleotide homopolymer-associated toxicity.

a, Boxplots of sgRNA $\log_2$ fold-change for all hDel-v2 sgRNAs (far left), sgRNAs containing G_4 nucleotide homopolymers (center left), sgRNAs containing A_4 nucleotide homopolymers (center right), and sgRNAs containing C_4 nucleotide homopolymers (far right). *p*-values were obtained by Mann–Whitney *U* test. Boxes extend from the first quartile to the third quartile. Whiskers extend from boxes by 1.5× the interquartile range.

b, Distribution of sgRNA $\log_2$ fold-change for all hDel-v2 sgRNAs (white) and sgRNAs containing G_4 nucleotide homopolymers (blue).
c, Fraction of sgRNAs significantly modifying cellular proliferation (FDR < 0.05) for all hDel-v2 sgRNAs (black) and G_4-containing sgRNAs (grey). Position 0 corresponds to the most PAM-distal position in the sgRNA spacer sequence ($G_4N_{16}NGG$). The number of sgRNAs with G_4 nucleotide homopolymers at the indicated position is labeled.
d, Boxplots of sgRNA $\log_2$ fold-change for sgRNAs containing G_4 nucleotide homopolymers at the indicated position (red, Mann–Whitney *U* test *p*-value < 0.05).
e, Fraction of sgRNAs significantly modifying cellular proliferation (FDR < 0.05) for all hDel-v2 sgRNAs (black) and C_4-containing sgRNAs (grey). The number of sgRNAs with C_4 nucleotide homopolymers at the indicated position is labeled.
f, Boxplots of sgRNA $\log_2$ fold-change for sgRNAs containing C_4 nucleotide homopolymers at the indicated position (red, Mann–Whitney *U* test *p*-value < 0.05).
g, Distribution of sgRNA $\log_2$ fold-change for all *MYC*- and *GATA1*-targeting sgRNAs[29] (white) and sgRNAs containing G_4 nucleotide homopolymers (blue).
h, Boxplots of sgRNA $\log_2$ fold-change for *MYC*- and *GATA1*-targeting sgRNAs containing G_4 nucleotide homopolymers at the indicated position (red, Mann–Whitney *U* test *p*-value < 0.05).

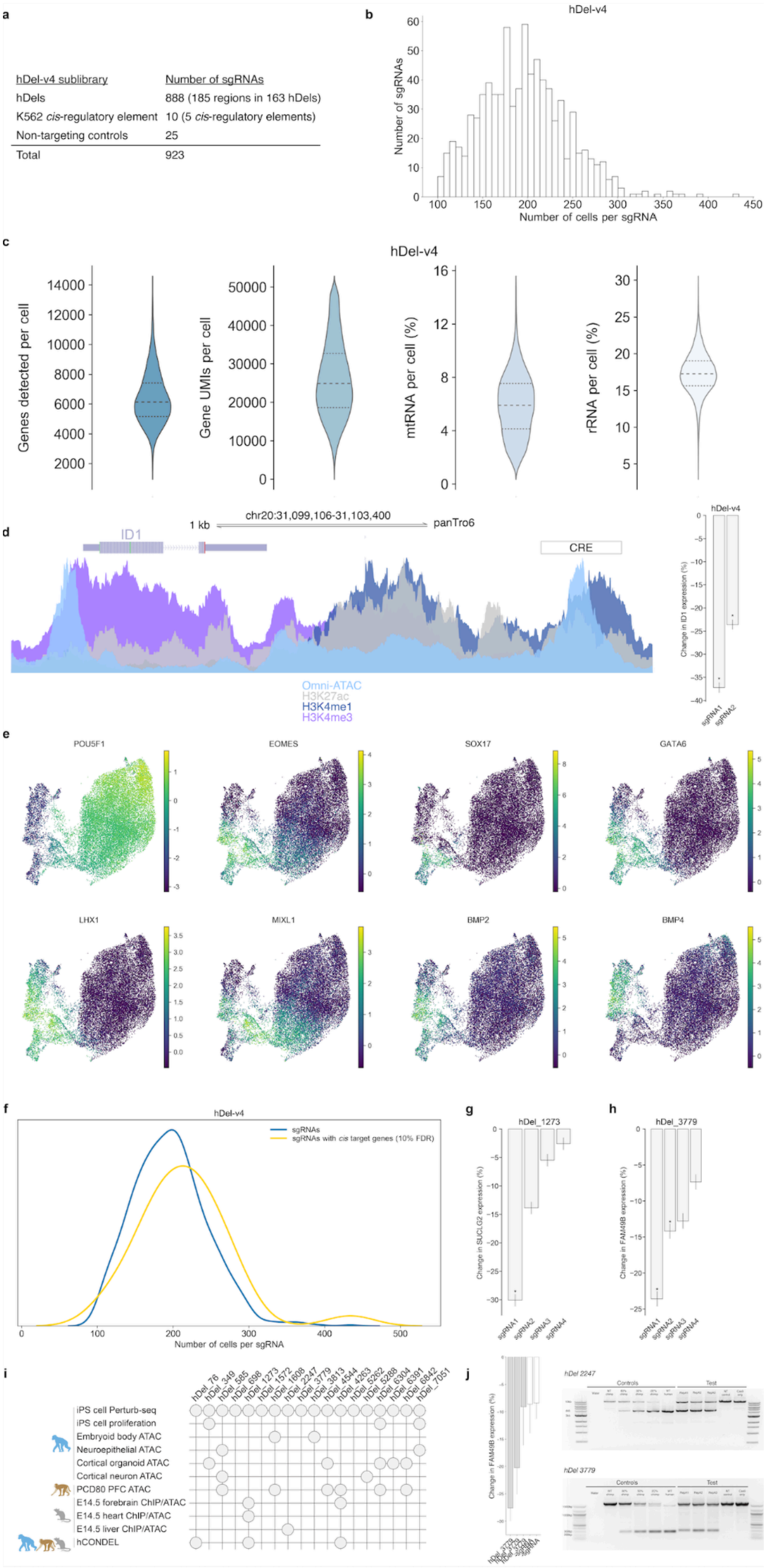
a
hDel-v4 sublibrary
Number of sgRNAs
hDels
888 (185 regions in 163 hDels)
K562 cis-regulatory element
10 (5 cis-regulatory elements)
Non-targeting controls
25
Total
923
b
hDel-v4
Number of sgRNAs
Number of cells per sgRNA
c
hDel-v4
Genes detected per cell
Gene UMIs per cell
mtRNA per cell (%)
rRNA per cell (%)
d
1 kb
chr20:31,099,106-31,103,400
panTro6
ID1
CRE
Omni-ATAC
H3K27ac
H3K4me1
H3K4me3
hDel-v4
Change in ID1 expression (%)
sgRNA1
sgRNA2
e
POU5F1
EOMES
SOX17
GATA6
LHX1
MIXL1
BMP2
BMP4
f
hDel-v4
sgRNAs
sgRNAs with cis target genes (10% FDR)
Number of cells per sgRNA
g
hDel_1273
Change in SUCLG2 expression (%)
h
hDel_3779
Change in FAM49B expression (%)
i
hDel_76
hDel_349
hDel_585
hDel_698
hDel_1273
hDel_1572
hDel_1608
hDel_2247
hDel_3779
hDel_3813
hDel_4544
hDel_4263
hDel_5262
hDel_5288
hDel_6304
hDel_6391
hDel_6842
hDel_7051
iPS cell Perturb-seq
iPS cell proliferation
Embryoid body ATAC
Neuroepithelial ATAC
Cortical organoid ATAC
Cortical neuron ATAC
PCD80 PFC ATAC
E14.5 forebrain ChIP/ATAC
E14.5 heart ChIP/ATAC
E14.5 liver ChIP/ATAC
hCONDEL
j
Change in FAM49B expression (%)
hDel 2247
Controls
Test
hDel 3779
Controls
Test

Supplementary Fig. 8 hDel-v4 Direct-capture Perturb-seq and Cas9-RNP validation.
a, hDel-v4 sublibraries.
b, Distribution of the number of cells per sgRNA.
c, Distribution of the number of genes detected (far left), gene UMIs (center left), percent mtRNA (center right), and percent rRNA (far right) per cell.
d, Omni ATAC-seq, H3K4me1, H3K4me3, and H3K27ac in C3624K and differential *ID1* expression for cells harboring the indicated *ID1 cis*-regulatory element[21]-targeting sgRNA (*FDR < 0.1).
e, UMAP projections colored by the normalized, log-transformed, and scaled expression of the indicated genes.
f, Distribution of the number of cells per sgRNA for all sgRNAs (blue) and sgRNAs with *cis* target genes (gold, FDR < 0.1).
g, Differential *SUCLG2* expression for cells harboring the indicated hDel_1273-targeting sgRNA (*FDR < 0.1).
h, Differential *FAM49B* expression for cells harboring the indicated hDel_3779-targeting sgRNA (*FDR < 0.1).
i, Intersection of hDels with identified *cis* target genes in chimpanzee iPS cells with ATAC-seq and ChIP-seq from chimpanzee, rhesus macaque, and mouse, and hCONDELs.
j, (left) Differential *FAM49B* expression (qRT-PCR) for cells edited with hDel_3779-targeting Cas9 RNPs (*FDR < 0.05). Bars represent independent polyclonal populations of chimpanzee iPS cells (C8861). (right) Gel electrophoresis for hDel_2247 (top) and hDel_3779 (bottom) amplicons from controls (dH_2O, chimpanzee gDNA, chimpanzee-human gDNA, and human gDNA) and experimental samples (replicate polyclonal populations electroporated with pairs of hDel-targeting Cas9 RNPs, -sgRNA paxGFP population, and Cas9-only population). 'Source data are provided as a Source Data file.

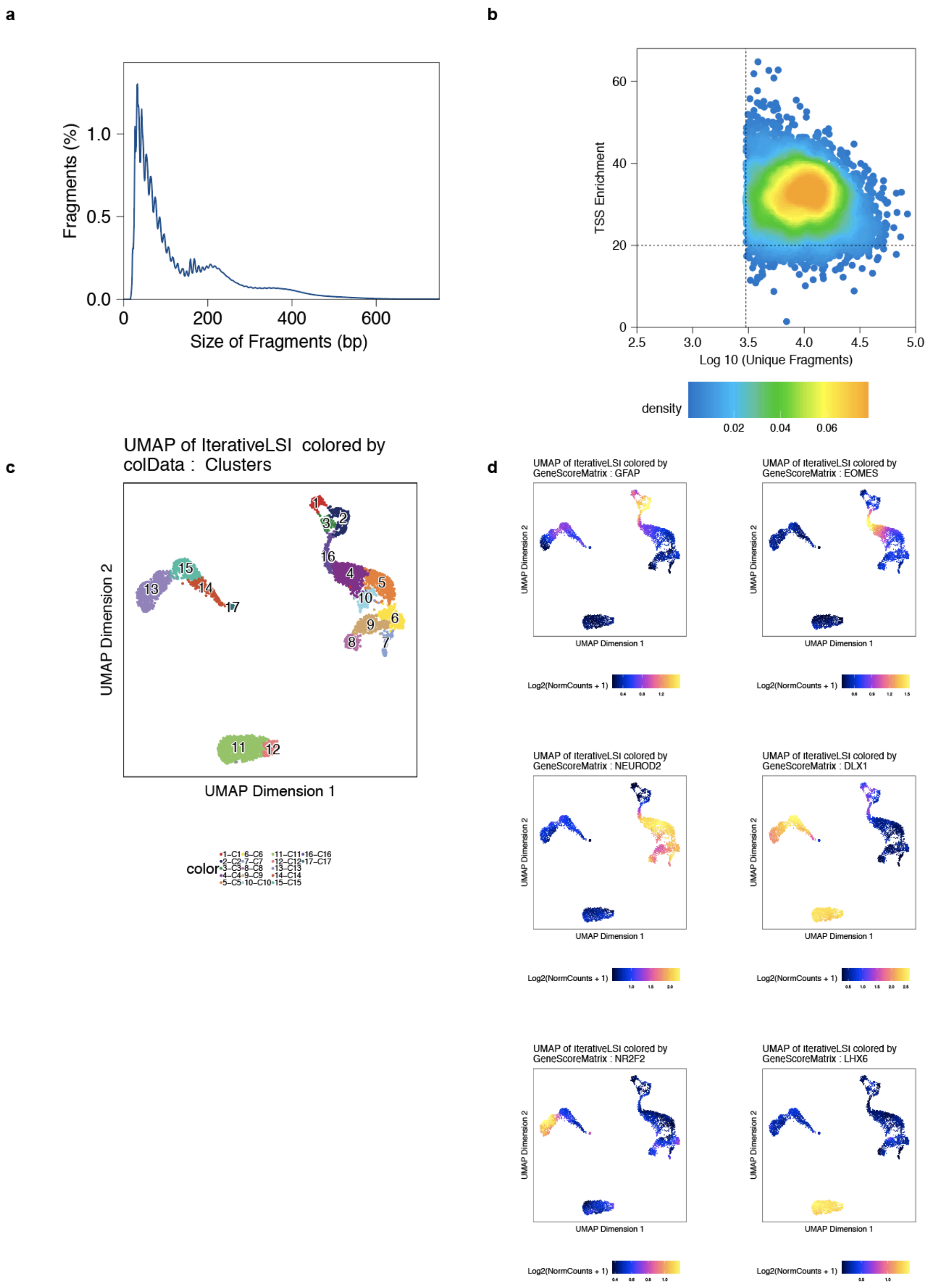

Supplementary Fig. 9 snATAC-seq of PCD80 rhesus macaque prefrontal cortex.

a, Distribution of snATAC-seq fragment sizes.

b, Density plot of the number of fragments and ArchR TSS enrichment score per cell.

c, UMAP projection colored by clusters (*n* = 17), including radial glia (RG, 1, 2, 3), intermediate progenitor cells-newborn excitatory neurons (IPC-nEN, 4, 16), excitatory neurons (EN, 5, 6, 7, 8, 9,

10), consisting of deep layer EN (8) and upper layer EN (6,9), and inhibitory neurons (IN, 11, 12, 13, 14, 15, 17), consisting of MGE-derived IN (11, 12), CGE-derived IN (13), LGE-derived IN (14, 15).
d, UMAP projection colored by the ArchR gene score of the indicated genes.

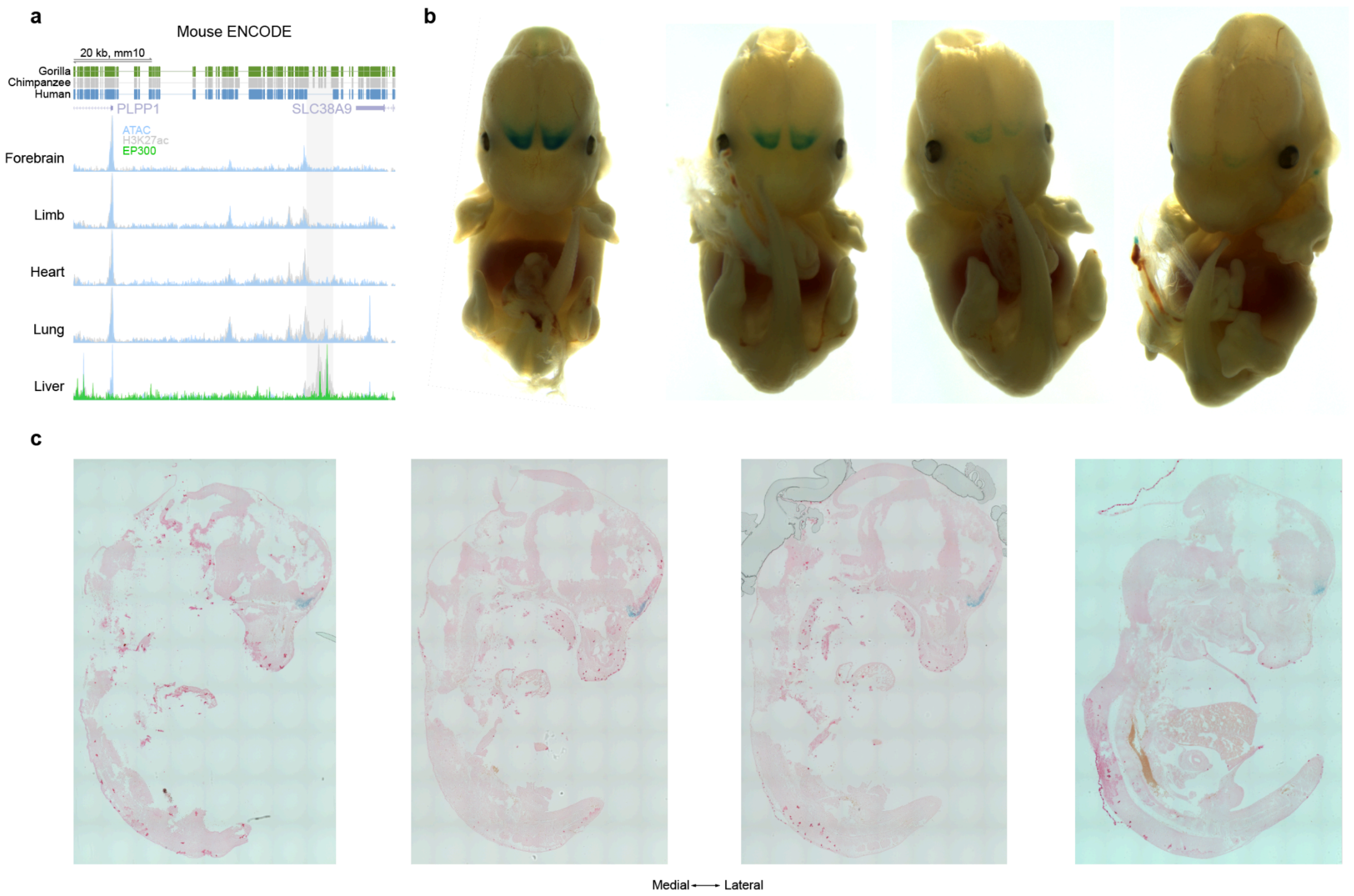

Supplementary Fig. 10 Tissue-specificity of hDel_2247.

a, Mouse ENCODE ATAC-seq, p300, and H3K27ac in E14.5 forebrain, limb, heart, lung, and liver tissues. Shaded region: hDel_2247 orthologous sequence.
b, E13.5 *hDel_2247*::*lacZ* mouse embryos stained for β-galactosidase (LacZ) activity. While the orthologous mouse sequence features epigenetic modifications (ATAC-seq, H3K27ac, p300) in the liver, the chimpanzee sequence drives *lacZ* expression in the olfactory bulb and anterior neocortex.
c, Sagittal sections showing *lacZ* expression.
