# Supplementary material for: Mapping cis- and trans-regulatory target genes of human-specific deletions
Source: Nat Commun. 2025 Dec 20;16:11380. doi: 10.1038/s41467-025-67424-x (PMC12727812; doi:10.1038/s41467-025-67424-x)
Supplement: Supplementary file 3 — Description of Additional Supplementary Files [file 41467_2025_67424_MOESM3_ESM.pdf]

Description of Additional Supplementary Files

File Name: Supplementary Data 1

Description: Human-specific deletion coordinates in the chimpanzee reference genome (panTro6).

File Name: Supplementary Data 2

Description: Tn5-accessible regions (Omni ATAC-seq) in chimpanzee iPS cells.

File Name: Supplementary Data 3

Description: pA-Tn5-accessible regions (CUT&Tag) in chimpanzee iPS cells.

File Name: Supplementary Data 4

Description: hDel-v1 sgRNA library and counts.

File Name: Supplementary Data 5

Description: hDel-v1 α-RRA.

File Name: Supplementary Data 6

Description: hDel-v2 sgRNA library and counts.

File Name: Supplementary Data 7

Description: hDel-v2 α-RRA.

File Name: Supplementary Data 8

Description: hDel-v3 sgRNA library.

File Name: Supplementary Data 9

Description: hDel-v3 *cis* sgRNA-gene pairs.

File Name: Supplementary Data 10

Description: hDel-v3 *trans* sgRNA-gene pairs.

File Name: Supplementary Data 11

Description: hDel-v4 sgRNA library.

File Name: Supplementary Data 12

Description: hDel-v4 *cis* sgRNA-gene pairs.

File Name: Supplementary Data 13

Description: Tn5-accessible regions (Omni ATAC-seq) in human, chimpanzee, and orangutan neural stem cells.
